# A clinical analysis of acute appendicitis with ileal perforation: a case report

**DOI:** 10.1093/jscr/rjaf857

**Published:** 2025-11-03

**Authors:** Shouda Yang, Jiexin Wu, Youxiang Guo, Jiang Fang, Bo Han, Yali Zhang, Baolin Wang

**Affiliations:** Department of General Surgery, The 63650 Military Hospital of PLA, Changjiang Road Sub-district, Shayibake District, Urumqi City, Xinjiang 841700, China; Department of Medical Statistics and Prevention, Chongqing Nanyu Middle School, Chenjiaqiao Subdistrict, Shapingba District, Chongqing 401331, China; Department of General Surgery, The 63650 Military Hospital of PLA, Changjiang Road Sub-district, Shayibake District, Urumqi City, Xinjiang 841700, China; Department of General Surgery, The 63650 Military Hospital of PLA, Changjiang Road Sub-district, Shayibake District, Urumqi City, Xinjiang 841700, China; Department of General Surgery, The 63650 Military Hospital of PLA, Changjiang Road Sub-district, Shayibake District, Urumqi City, Xinjiang 841700, China; Department of Disease Prevention and Control, The 63650 Military Hospital of PLA, Xinjiang 841700, China; Department of General Surgery, The 63650 Military Hospital of PLA, Changjiang Road Sub-district, Shayibake District, Urumqi City, Xinjiang 841700, China

**Keywords:** acute appendicitis, ileal perforation, acute abdominal conditions

## Abstract

This article reports a rare case of a patient with acute appendicitis in whom ileal perforation was incidentally found during surgery. The patient was admitted with typical symptoms of right lower quadrant abdominal pain and was diagnosed with acute appendicitis based on physical examination, laboratory tests, and CT imaging findings. An emergency laparoscopic exploration was performed. During the operation, two perforations in the ileum, 10 cm away from the ileocecal junction, were unexpectedly discovered. The patient underwent perforation repair combined with appendectomy. Postoperative recovery was uneventful, and no complications were observed during a 2-month follow-up. This case highlights the clinical importance of considering the possibility of coexisting acute abdominal conditions in patients with appendicitis and emphasizes the significance of systematic intraoperative exploration, providing a reference for the diagnosis and treatment of similar cases.

## Introduction

Appendicitis, as one of the most common acute abdominal emergencies in general surgery, carries a lifetime risk of ~7%–8% [1]. Studies indicate that 20%–30% of acute appendicitis patients present with atypical clinical manifestations, significantly complicating diagnosis and management [2]. Despite continuous advancements in diagnostic and therapeutic techniques, missed diagnoses may lead to concurrent intra-abdominal pathologies such as ileal perforation, which can arise from diverse etiologies including infectious (e.g. typhoid), inflammatory (e.g. Crohn’s disease), neoplastic, or traumatic causes [3]. This article reports a special case initially diagnosed with acute appendicitis, in which an ileal perforation was discovered during surgery, and discusses its clinical features and management strategies.

## Clinical Description

A 20-year-old male patient of Asian descent was admitted to the hospital with a chief complaint of “persistent distension and pain in the right lower abdomen for 2 days, worsening for 6 h.” The patient denied recent travel to typhoid-endemic regions or exposure to infectious diseases. No family history of inflammatory bowel disease (IBD) or prior abdominal surgeries was reported. Physical examination revealed tenderness (+) at McBurney’s point, rebound tenderness (+), and a positive colon inflation test. Laboratory findings: WBC 11.67 × 10^9^/l, neutrophils 69.2%, C-reactive protein (CRP) 20.44 mg/l. Abdominal computed tomography (CT) demonstrated an 8-mm dilated appendix with a fecalith, periappendiceal fat stranding, and minimal free fluid in the right iliac fossa without evidence of pneumoperitoneum. Preoperative diagnosis: acute appendicitis.

### Surgical procedure

**Figure 1:**
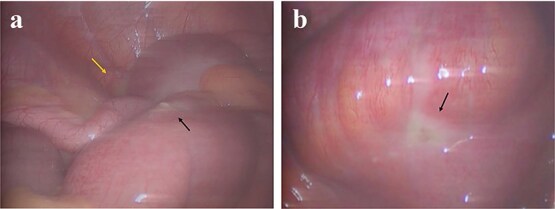
Intra-operative laparoscopic findings. (a) Approximately 15 ml of purulent fluid (yellow arrow) and the first ileal perforation (black arrow) in the right iliac fossa. (b) A second ileal perforation (black arrow) identified in an adjacent loop.

**Figure 2:**
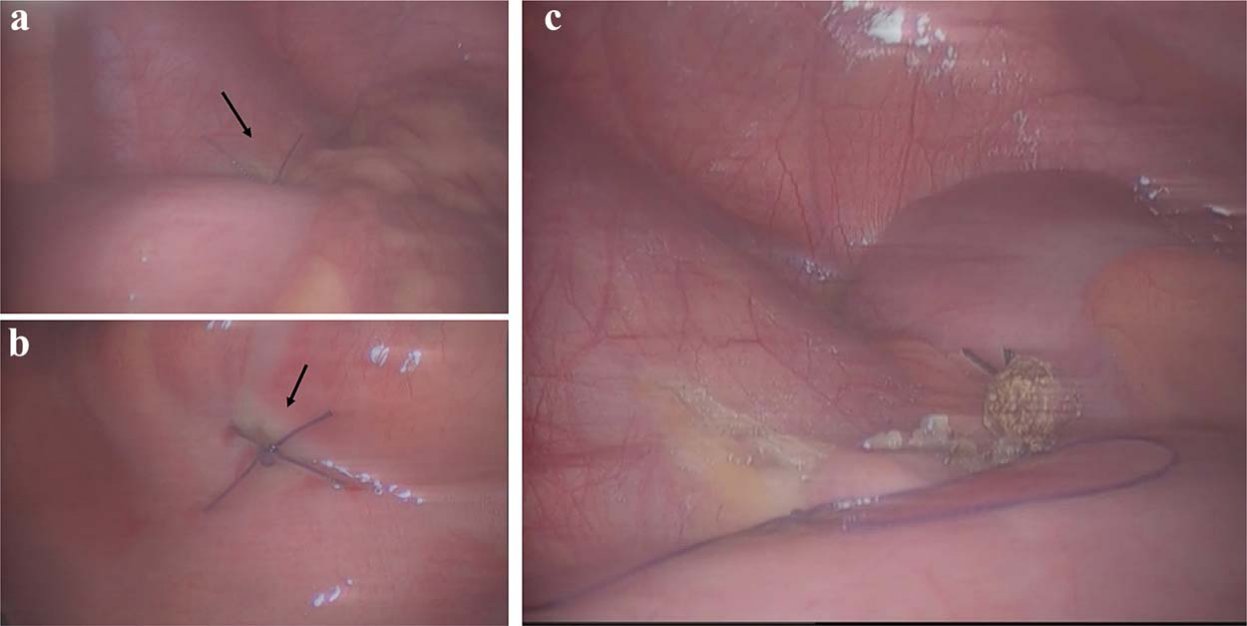
Intra-operative management. (a and b) Laparoscopic full-thickness suture closure (black arrow) of the two ileal perforations. (c) Completed laparoscopic appendectomy.

A laparoscopic exploration via a three-port approach was performed under general anesthesia. Intraoperatively, 15 ml of purulent exudate was found in the right iliac fossa, and two adjacent ileal perforations measuring 0.5 cm each were identified 10 cm proximal to the ileocecal valve, located 3 cm apart within an edematous bowel segment (Fig. 1). The appendix measured ~8 cm in length, was congested and swollen, without gangrene or perforation. Given the proximity of perforations and absence of pre-existing bowel pathology, the operative findings suggested secondary ileal perforation due to localized peritonitis from appendiceal inflammation. The procedure included double-layer suture repair of the ileal perforations and appendectomy (Fig. 2), with placement of a peritoneal drainage tube.

### Postoperative outcome

Postoperatively, the patient received anti-infective treatment with cefozopran sodium in combination with tinidazole, along with nutritional support. Intestinal peristalsis resumed on the fifth postoperative day, the drainage tube was removed on the sixth day, and the patient was discharged on the seventh day. The pathology confirmed acute uncomplicated appendicitis. Capsule endoscopy was advised to exclude occult Crohn’s disease but was declined by the patient.

## Discussion

Acute appendicitis is the most common cause of emergency abdominal surgery for a surgical acute abdomen [4]. Recent guidelines, including the right iliac fossa treatment (RIFT) study and WSES Jerusalem guidelines, emphasize the role of early CT imaging in reducing diagnostic uncertainty [5]. CT demonstrates high sensitivity (94%) and specificity (96%) for appendicitis diagnosis [6], though its limitations in detecting early perforations—as seen in this case—highlight the necessity for systematic laparoscopic exploration in equivocal scenarios [7].

Key clinical insights from this case include: (A) The operative success likely resulted from timely intervention before widespread contamination, rather than inherent superiority of primary repair. In delayed presentations with established peritonitis, diverting stomas may be preferable. (B) The treatment primarily involves management of the perforation site and systemic administration of antibiotics. In most cases, simple suture of the perforation is sufficient. However, if multiple perforations are present in a single segment of the intestine, partial resection and anastomosis of the bowel may be more appropriate [8]. (C) While IBD was considered in differential diagnosis, the absence of histological or endoscopic evidence precludes definitive attribution, underscoring the need for thorough postoperative evaluation.

## Conclusion

Acute appendicitis complicated by intestinal perforation is clinically rare and prone to missed diagnosis. Clinicians should: (A) consider enhanced CT protocols to improve perforation detection; (B) rigorously explore all abdominal quadrants during surgery, particularly in young patients with unexplained inflammatory markers; (C) reserve conclusions about etiology (e.g. Crohn’s disease) until objective evidence is obtained. Multidisciplinary collaboration and meticulous surgical techniques remain paramount for optimizing outcomes.

## References

[1] Stewart B, Khanduri P, McCord C, et al. Global disease burden of conditions requiring emergency surgery. Br J Surg 2014;101:e9–22. 10.1002/bjs.9329 24272924

[2] Bhangu A, Søreide K, Di Saverio S, et al. Acute appendicitis: modern understanding of pathogenesis, diagnosis, and management. Lancet. 2015;386:1278–87. 10.1016/S0140-6736(15)00275-5 26460662

[3] RIFT Study Group . Right iliac fossa treatment (RIFT) study: protocol for an international, multicentre, prospective observational study. BMJ Open 2018;8:e017574. 10.1136/bmjopen-2017-017574 PMC5780718 29331965

[4] Cheluvappa R, Thomas DG, Selvendran S. The role of specific chemokines in the amelioration of colitis by appendicitis and appendectomy. Biomolecules 2018;8:1–13. 10.3390/biom8030059 30037025 PMC6165111

[5] Di Saverio S, Podda M, De Simone B, et al. Diagnosis and treatment of acute appendicitis: 2020 update of the WSES Jerusalem guidelines. World J Emerg Surg 2020;15:27. 10.1186/s13017-020-00306-3 32295644 PMC7386163

[6] van Randen A, Bipat S, Zwinderman AH, et al. Acute appendicitis: meta-analysis of diagnostic performance of CT and graded compression US related to prevalence of disease. Radiology 2008;249:97–106. 10.1148/radiol.2483071652 18682583

[7] Moris D, Paulson EK, Pappas TN. Diagnosis and management of acute appendicitis in adults: a review. JAMA 2021;326:2299–311. 10.1001/jama.2021.20502 34905026

[8] Singh G, Dogra BB, Jindal N, et al. Non-traumatic ileal perforation: a retrospective study. J Family Med Prim Care 2014;3:132–5. 10.4103/2249-4863.137633 25161970 PMC4139993

